# A biomechanical comparison of su5ture with a lateral plate and cerclage wire and suture constructs for lesser tuberosity osteotomy repair in stemless anatomic total shoulder arthroplasty

**DOI:** 10.1016/j.jsea.2026.100099

**Published:** 2026-09-30

**Authors:** Kyle B. Christy, Randall A. Arroyo, Mitchell S. Kirkham, Peter N. Chalmers, Robert Z. Tashjian, Heath B. Henninger, Christopher D. Joyce, Maarouf A. Saad

**Affiliations:** a Department of Orthopaedics, University of Utah, Salt Lake City, UT, USA; b School of Medicine, University of California, Riverside, CA, USA; c Department of Surgery, University of Washington, Seattle, WA, USA; d Department of Biomedical Engineering, University of Utah, Salt Lake City, UT, USA; e Department of Mechanical Engineering, University of Utah, Salt Lake City, UT, USA; f Department of Orthopaedic Surgery and Biomedical Engineering, University of Tennessee Health Sciences Center-Campbell Clinic, Memphis, TN, USA

**Keywords:** Shoulder, Total shoulder arthroplasty, Lesser tuberosity osteotomy, Cyclic testing, Failure testing, Suture, Wire, Plate

## Abstract

**Background:**

There are limited biomechanical data on lesser tuberosity osteotomy (LTO) repair techniques in the setting of stemless implants. The risk of LTO nonunion from inadequate compression and/or resultant bone fragment micromotion may be mitigated by biomechanically improved repair techniques. The purpose of this study was therefore to evaluate the biomechanical properties of a suture with a lateral plate construct (suture-plate) when compared to a cerclage wire construct (wire-suture) in the setting of stemless implants.

**Methods:**

Thirteen matched pairs of human cadaveric shoulders were dissected and the lesser tuberosity osteotomized. In each pair, the LTO was repaired with a suture-plate construct on one side and a wire-suture construct on the contralateral side. Both constructs utilized suture fixation through the suture slots onto the stemless component. Using a custom subscapularis cryoclamp, each specimen was cycled from 10 to 100 N at 1 Hz 1,000 times. Cyclic gap formation was recorded using a digital video system. Each specimen was then loaded to failure with load, displacement, construct stiffness, and mode being recorded. Statistical analyses compared the paired suture-plate and wire-suture constructs.

**Results:**

There were no differences between constructs in LTO dimensions, cyclic gap formation, failure load, or failure displacement between constructs (*P* ≥ .133). The suture-plate construct was approximately 20% stiffer than the wire-suture construct (*P* = .033). The most frequent failure mode in the suture-plate group was due to suture tearing (5/13, 38%), whereas the wire-suture group's most common failure mode was due to fractures through the bone tunnels created for the wires (10/13, 77%).

**Conclusion:**

In this controlled laboratory study, there were no differences in gap formation and failure load between the suture with a lateral plate and cerclage wire constructs, suggesting that there may be no differences in time-zero fixation, and environments for *in vivo* LTO healing rates may exist. However, the decreased stiffness and increased number of bone tunnel failures seen in the cerclage wire**-**suture construct suggest that sutures with plate constructs may be more resilient at higher loads, and in patients with weaker bone, lower the risk for fracturing of the bone tunnels. While clinical data are needed to definitively prove this observation, LTO repair with a suture and lateral plate construct may provide a biomechanically superior repair to a cerclage wire**-**suture repair in the setting of stemless anatomic total shoulder arthroplasty.

Subscapularis takedown and subsequent repair, which is required to expose the glenohumeral joint during total shoulder arthroplasty with a deltopectoral approach, is performed via 3 primary techniques: tenotomy, subscapularis peel, or lesser tuberosity osteotomy (LTO).^24^ Preserved subscapularis integrity, bone-to-bone healing, which is easily monitored via axillary radiographs, and prevention of suture pull-through are theoretical advantages provided by an LTO.^10^, ^11^, ^24^, ^25^, ^26^ There are conflicting reports of superiority when comparing the post-operative clinical outcome scores, physical exam test results, and subscapularis tear incidence of patients who received a tenotomy or peel to those who received an LTO.^4^, ^6^, ^11^, ^27^ Additionally, tenotomy and peel techniques have shown increased cyclic gapping and decreased failure load during biomechanical testing compared to LTO.^9^, ^17^, ^25^, ^28^ *In vivo*, increased cyclic gapping compromises subscapularis healing, and lower failure loads suggest an increased risk for failure of the repair construct.^19^

Multiple methods for suture-based LTO repair constructs are described. Traditional transosseous repairs involve suture passage from tunnels lateral to the osteotomy site, through the medullary canal, exiting medial to the osteotomy site, through the tendon of the subscapularis, and tied over the LTO to create a compression effect.^7^, ^17^, ^25^ Another technique consists of inverted mattress sutures placed through the subscapularis tendon, then passed transosseously from the bicipital groove to the lateral aspect of the greater tuberosity and tied over a plate creating a tension band effect.^10^, ^11^, ^30^ While these constructs were developed for stemmed humeral components, another suture construct has been described that consists of both compression and tension band sutures for use in stemless implants.^16^

In a biomechanical study, a dual-row compression suture construct displayed was compared to a tension band construct and demonstrated less cyclic gapping and higher loads to failure in stemmed implants.^14^ In another study, a compression suture construct had a lower ultimate failure loads when compared to a tensionable suture tape construct in stemmed implants.^7^ In the setting of stemless implants, a construct using transosseous sutures tied with a racking hitch stitches combined with inverted mattress sutures tied over a lateral plate (suture-plate group in the present study) displayed less cyclic gapping than a suture anchor construct and higher failure loads than a suture anchor construct as well as a tensionable cortical button construct.^16^ Transosseous wires have been used clinically with reported 93% LTO healing rates when used with a stemless implant.^20^, ^26^ Currently, no biomechanical studies have been performed evaluating the time zero strength of LTO repairs using transosseous wires.

Therefore, the purpose of this study was to evaluate the biomechanical properties of 2 complete LTO repair constructs, a construct using transosseous sutures tied with a racking hitch stitches combined with inverted mattress sutures tied over a lateral plate (suture-plate) to a transosseous cerclage wire-suture (wire-suture) construct in the settling of stemless implants. We hypothesized the suture-plate construct would have decreased cyclic gapping and a greater ultimate failure load when compared to the wire-suture construct, potentially translating to improved LTO healing.

## Methods

### Specimen preparation

Thirteen pairs of fresh-frozen human cadaveric shoulders were obtained (7 male, 6 female; age: 67 ± 14 years; body mass index: 23.8 ± 6.9 kg/m^2^; United Tissue Network, Norman, OK, USA, and ScienceCare, Phoenix, AZ, USA). All specimens were thawed at room temperature, and subsequently, the glenohumeral joint was disarticulated with the subscapularis muscle belly and tendon intact on the lesser tuberosity. The supraspinatus and infraspinatus muscle bellies were dissected, leaving the tendon stumps intact on the greater tuberosity. Throughout dissection, specimens were kept moist with normal saline. After dissection, specimens were wrapped in normal saline-soaked towels and frozen until testing. For each pair, a coin flip determined which side would receive the suture-plate (6 right, 7 left) or wire-suture (7 right, 6 left) construct.

Prior to preparation by fellowship trained shoulder and elbow surgeon (M.S.), specimens were thawed at room temperature for 24 hours. For both constructs, the LTO and implantation of the stemless humeral component (Equinoxe Stemless Shoulder; Exactech Inc., Gainesville, FL, USA) were performed in the same manner. First, any excess capsular material was dissected, the rotator interval incised, and the biceps tendon transected. Using a 2.5 cm oscillating saw blade, the lesser tuberosity was osteotomized from the base of the bicipital groove to the anatomic neck. Digital calipers (accuracy: ±0.02 mm) were used to measure the LTO fragment: proximal-distal length, cut surface width perpendicular to length, and greatest thickness from cortical to cancellous bone. At the anatomic neck, a free hand cut was performed, osteotomizing the humeral head. Next, using the standard manufacturer guidelines, a guide pin was placed into the humeral metaphysis, and the cancellous bone was reamed and subsequently punched.

### Suture with a lateral plate construct

The LTO was repaired using the suture with a lateral plate construct as previously described.^16^ After osteotomizing the lesser tuberosity, 3 equally spaced 2.0 mm tunnels were drilled lateral to the bicipital groove. The free ends of #2 braided high-tensile strength sutures (FiberWire; Arthrex, Inc., Naples, FL, USA) were passed in a looped fashion through the 2 proximal drill tunnels and then into the suture slots of the humeral component prior to being passed back through the humerus medial to the osteotomy site. A third suture was passed through the most distal lateral tunnel through a medial tunnel to create a bone bridge prior to performing the final impaction of the implant into the metaphysis. Using a free needle, the free ends were passed through the tendon insertion of the subscapularis, tied as Nice knots,^2^ and tensioned by hand. Then, 2 #5 braided high-tensile strength sutures (FiberWire) were passed in an inverted mattress configuration through the subscapularis tendon just medial to the #2 sutures. Three 2.0 mm tunnels were drilled from the base of the bicipital groove to the greater tuberosity, approximately 1 cm posterior to the bicipital groove. The #5 sutures were passed through these tunnels, then through a 2.7 mm ¼ tubular plate, tied in a simple fashion, and tensioned by hand (Figs. 1 and 2).

**Figure 1:**
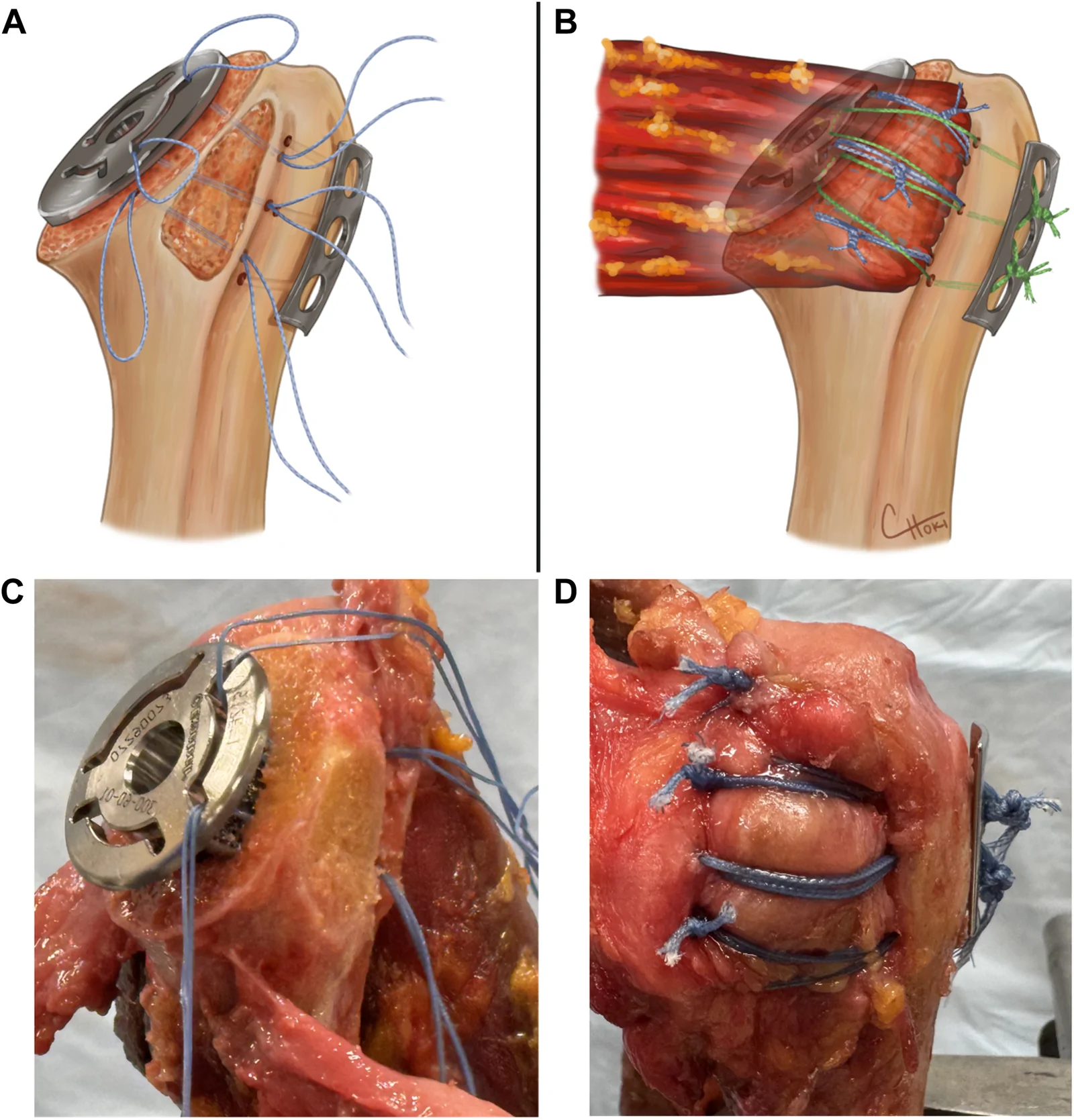
Suture with a lateral plate construct. Illustration (**A**) and photograph (**C**) of the #2 sutures passing from the bicipital groove and through the suture slots of the humeral component. Illustration (**B**) and photograph (**D**) of the completed construct. This figure was published in *Journal of Shoulder and Elbow Surgery*, Kirkham et al., Lesser tuberosity osteotomy repair in stemless anatomic shoulder arthroplasty: a biomechanical analysis of repair techniques,^16^ Copyright Elsevier (2026).

**Figure 2:**
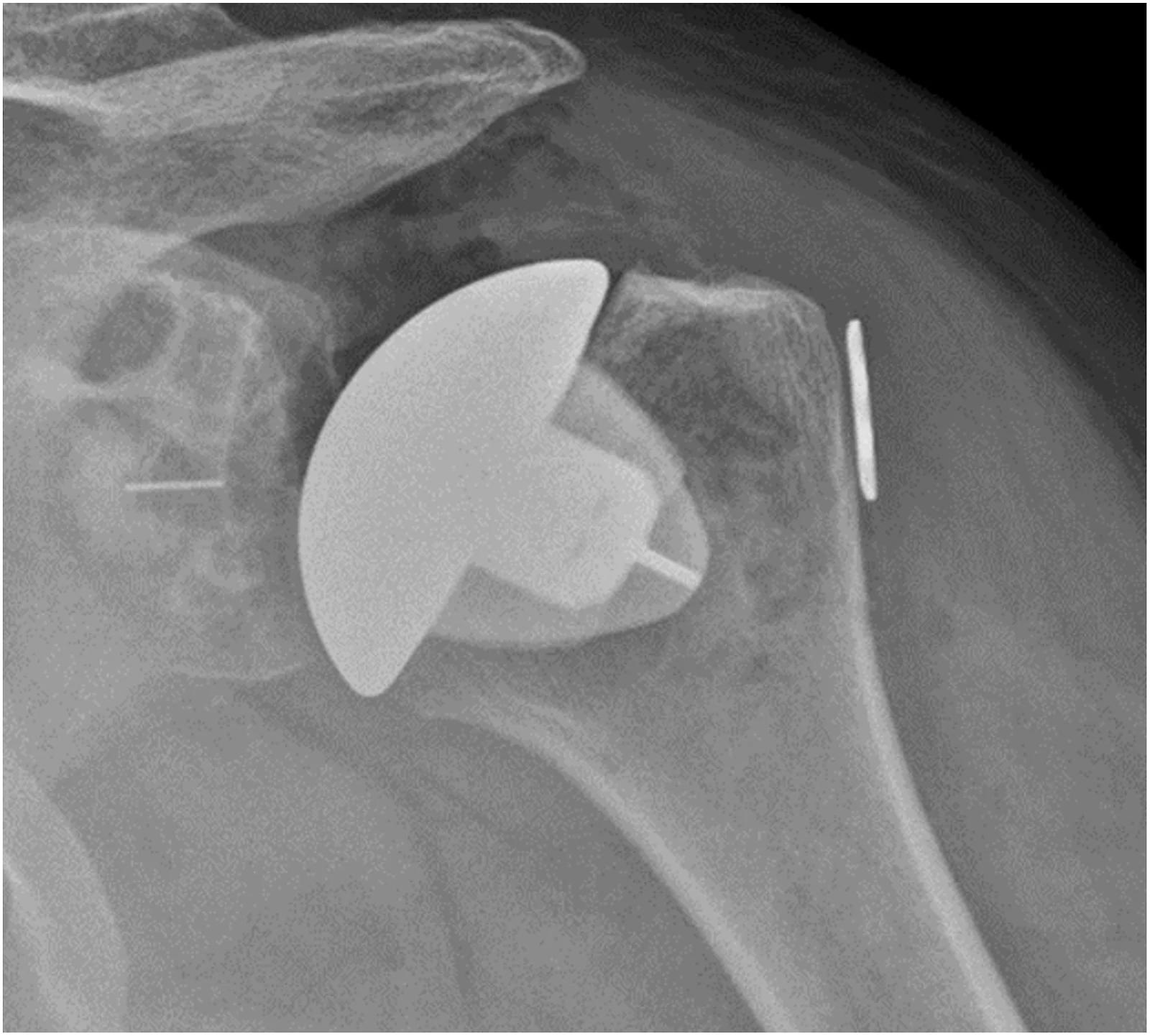
Suture with a lateral plate radiograph. Grashey view of an LTO repaired with a suture with a lateral plate. *LTO*, lesser tuberosity osteotomy.

### Transosseous cerclage wire and suture construct

The LTO was repaired with the wire-suture construct as previously described.^20^, ^26^ After osteotomizing the lesser tuberosity, 4 2.0 mm tunnels were drilled: 1 proximal and 1 distal separated by 2 cm on the lateral proximal humerus just lateral to the osteotomy site, and 1 proximal and 1 distal separated by 2 cm just medial to the osteotomy site at the level of the calcar. The free ends of the braided high-tensile strength #5 suture loop (FiberWire) and 18-gauge stainless steel wire were passed from the lateral distal tunnel out the medial distal tunnel. This was repeated with a second #5 suture loop and 18-gauge stainless steel wire for the proximal tunnels, with the proximal suture loop and wire additionally being passed through the suture slots of the humeral component prior to exiting the medial tunnel. The free ends of the suture and the wires were passed through the insertion of the subscapularis tendon. The sutures were tied as Nice knots,^2^ being tensioned by hand, and the wire ends were twisted together 5 times, tightened on the lesser tuberosity, and wire ends were removed leaving a ∼1 cm segment of twisted wire (Figs. 3 and 4).

**Figure 3:**
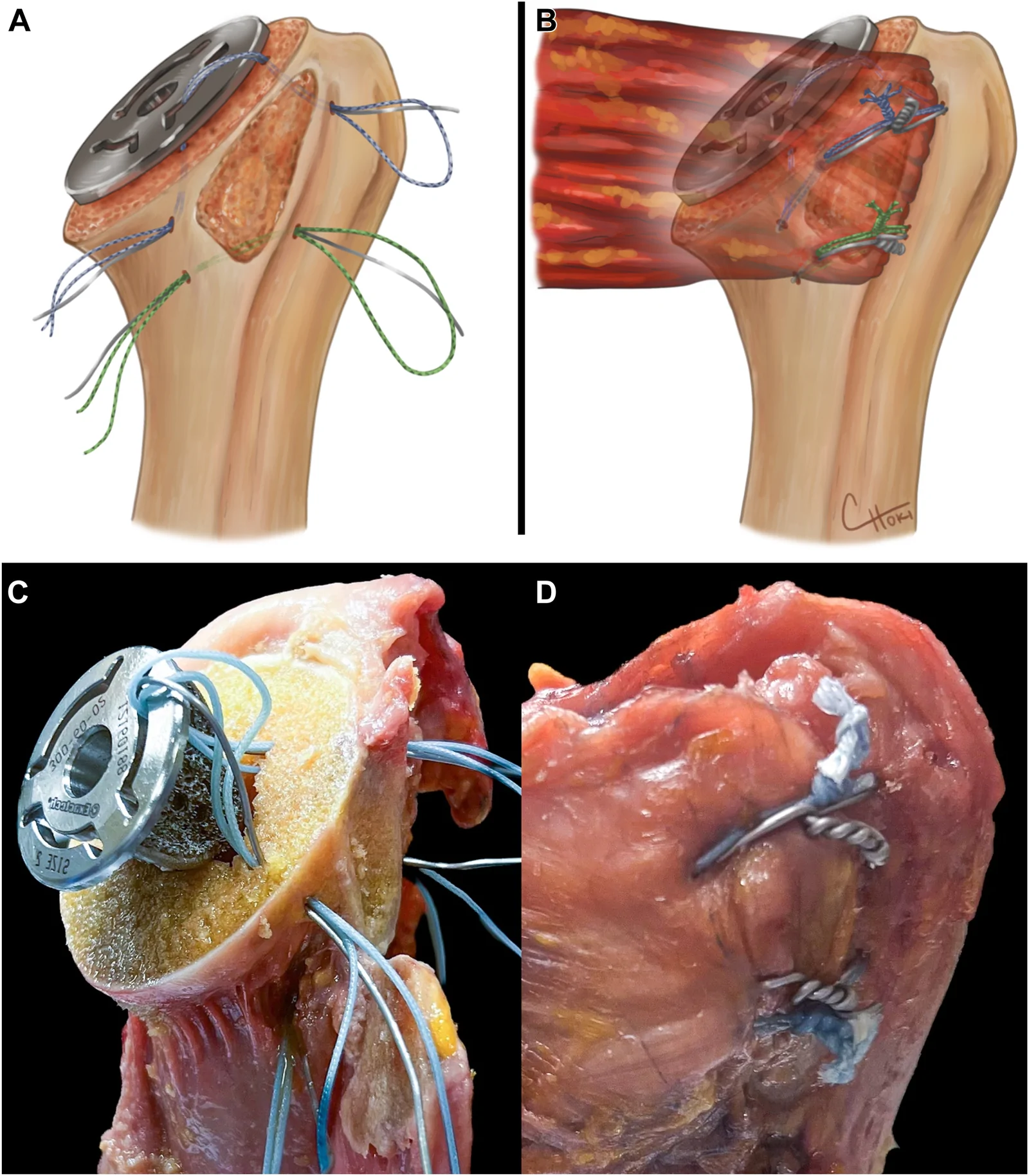
Transosseous cerclage wire and suture construct. Illustration (**A**) and photograph (**C**) of the looped #5 sutures and passing from the bicipital groove, through the metaphysis, and exiting medial to the osteotomy site, and in the case on the top suture and wire, passing through the suture slot on the humeral component. Illustration (**B**) and photograph (**D**) of the completed construct.

**Figure 4:**
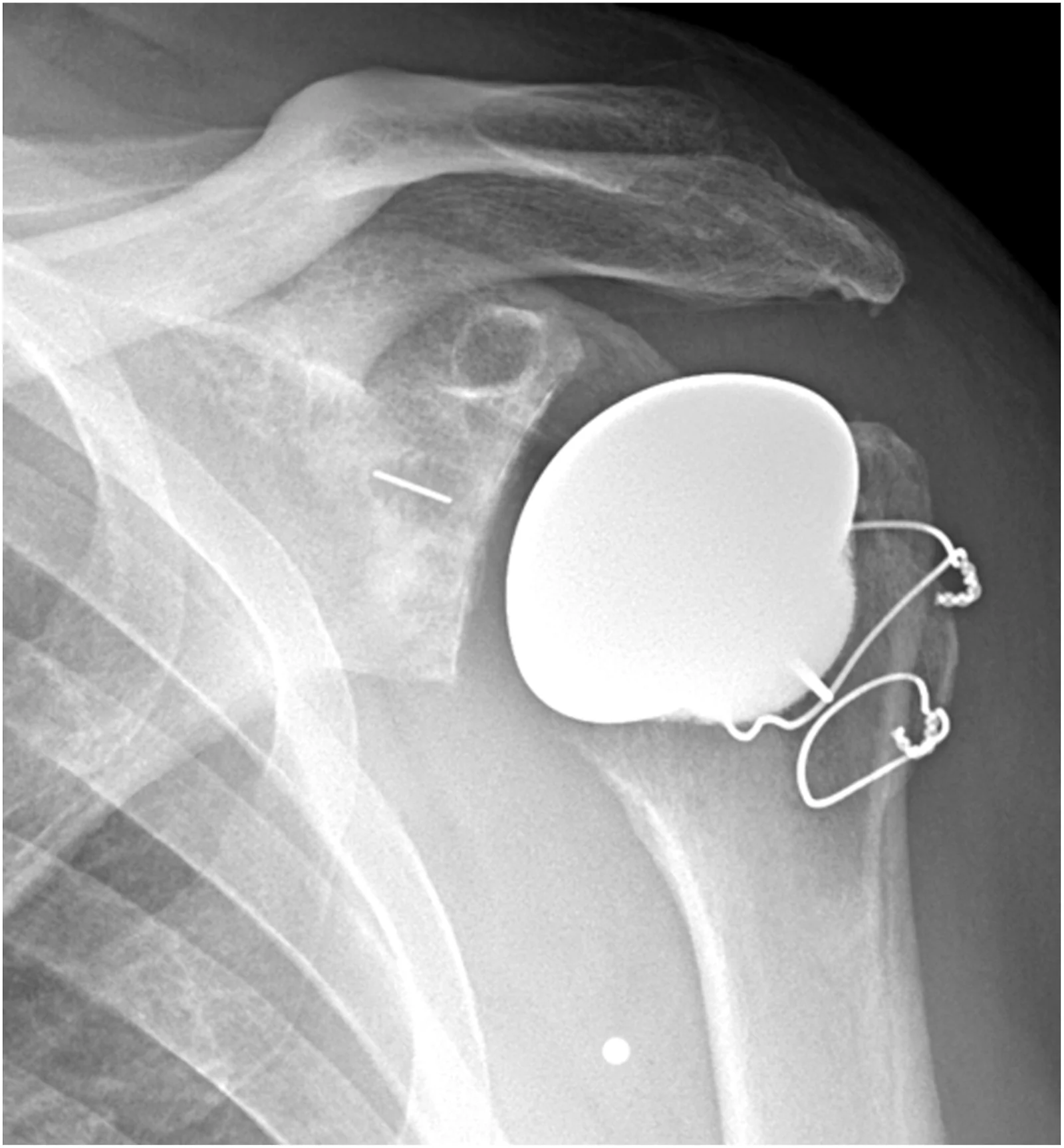
Transosseous cerclage wire and suture radiograph. Grashey view of an LTO repaired with a transosseous cerclage wires. *LTO*, lesser tuberosity osteotomy.

A figure-of-8 suture was used for both constructs to close the release of the rotator interval between the supraspinatus and subscapularis tendons.

### Mechanical testing protocol

A testing protocol was adapted from a prior study of LTO repair biomechanics.^16^ Humeri were horizontally mounted onto the machine (1331 Load Frame, model 8800 controller; Instron, Norwood, MA, USA), and the subscapularis muscle belly was secured in line with the vertical actuator in a custom cryoclamp. In the present study, the edge of a metal plate mounted onto a tower contacted the humeral component to simulate the compressive resistance provided by the glenoid component of the stemless arthroplasty in glenohumeral joint (Fig. 5).

**Figure 5:**
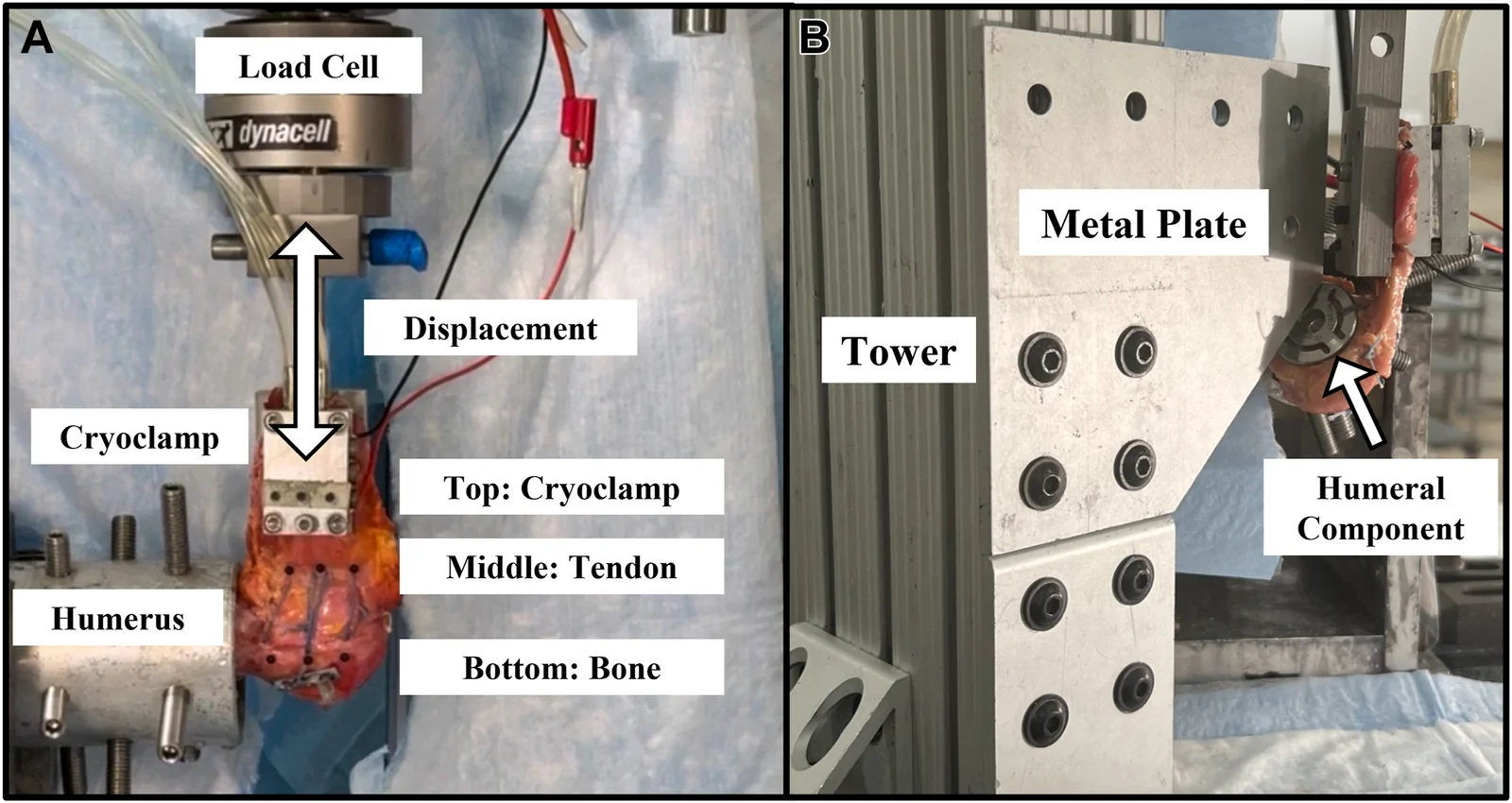
Testing apparatus: (**A**) The muscle belly of the subscapularis was fixed in a custom cryoclamp that was attached to the load cell and was cycled parallel to the muscle fibers. Motion tracking beads were placed on the cryoclamp, subscapularis tendon medial to the construct but lateral to the musculotendinous junction and on the bone just lateral to the construct. Differences in bead pairs between the top 2 rows represent tissue elongation and between the bottom 2 rows, construct gapping. (**B**) A metal plate attached to a tower provided resistance to the humeral component to simulate glenohumeral contact forces.

To normalize starting conditions, a 10 N preload was applied to the repair and held for 60 seconds. Next, using a triangle waveform, the specimens were cycled between 10-100 N, 1,000 times at 1 Hz. After cycling, the actuator was displaced at 1 mm/s until construct failure occurred. Throughout the test, a 1-kN load cell (Dynacell, model 2527-130; Instron) measured applied force, and a normal saline solution spray was used to keep the tissue hydrated. In the event that failure loads exceeded the capacity of the load cell, an overload limit ended the test.

### Marker tracking and data collection

A digital video camera (Prosilica GC1350 Gigabit Ethernet, 1360 × 1024-pixel resolution; Allied Vision Technologies, Exton, PA, USA) (0.1% field-of-view accuracy; <0.05 mm after calibration) was used for marker tracking with semi-automated software (Digital Motion Analysis System [DMAS], v6.5; Spicatek, Fox Island, WA, USA), following a protocol from prior studies.^1^, ^16^, ^18^, ^29^

Using cyanoacrylate, 3 rows of 3 2 mm markers were placed (Fig. 5, A). Construct gapping was represented by the average bead pair distance between the bone and tendon rows, while tissue elongation was represented by the average bead pair distance between the tendon and clamp rows. Construct gapping and tissue elongation were taken at 1, 10, 50, 100, 200, 300, 400, 500, and 1,000 cycles as an average over 3 cycles. If a bead was unable to be tracked, that bead pair was excluded from analysis at that cycle. A cubic spline interpolation between the data points was used to create continuous data at each cycle.

Load and bead displacement were continuously recorded throughout the failure test. Construct failure was defined as the first peak load recorded prior to a rapid decrease in load magnitude, regardless if any subsequent peak(s) were greater. If a specimen did not fail prior to reaching a supraphysiological 1,050.0 N load cell limit, a failure load of 1,050.0 N and construct displacement at said load were used for statistical analysis. The slope of the load-displacement curve immediately preceding its slope inflection point (ie, yield) before failure defined construct stiffness (N/mm), which was calculated using a custom script in MATLAB R2026a (MathWorks; Natick, MA, USA). Observed failure modes were also recorded.

### Statistical analysis

An *a priori* power analysis was performed based on prior work.^16^ To achieve 0.80 power using a 2-tailed, matched-pair power analysis (G∗Power, v3.1.9.7),^8^ 13 pairs were needed to calculate a 1.1 ± 1.0 mm mean difference in cyclic gapping and 100.0 ± 90.0 N mean difference in failure load. Continuous construct gapping during cyclic testing was compared between suture-plate and wire-suture groups using statistical parametric mapping for a paired *t*-test (version M.0.4.11; www.spm1d.org).^21^ Statistical parametric mapping allows for statistical consideration of variability across the cyclic test using a random effects analysis to account for repeated measures. All other analyses were performed in SPSS (v31, IBM, Armonk, NY, USA). The LTO dimensions, tissue elongation, and failure metrics were compared using a paired *t*-test. Mean differences were presented as mean ± standard deviation (95% confidence interval). McNemar's test was used for a secondary analysis to compare the frequency of a bone tunnel fracture failure between groups. Statistical significance was defined as *P* ≤ .050.

## Results

There were no differences in the dimensions of the LTO between repair constructs (Table I). There were no differences between constructs for gapping at any cycle (Fig. 6) or tissue elongation. There were no differences in failure load (mean difference: 87.7 ± 196.0 [-30.7, 206.1]) or displacement (mean difference: −1.5 ± 10.1 [−8.1, 5.1]) between constructs (Table II). The suture-plate construct was approximately 20% stiffer (mean difference: 20.7 ± 31.0 [1.9, 39.4]) than the wire-suture construct (*P* = .033) (Table II).

**Table I:** LTO dimensions (mean ± SD).

Variable	Suture	Wire	*P* value
Length (mm)	25.8 ± 5.6	24.4 ± 3.0	.403
Width (mm)	19.0 ± 4.3	18.7 ± 3.2	.656
Thickness (mm)	8.2 ± 2.0	7.8 ± 1.5	.406

*LTO*, lesser tuberosity osteotomy; *SD*, standard deviation.

**Figure 6:**
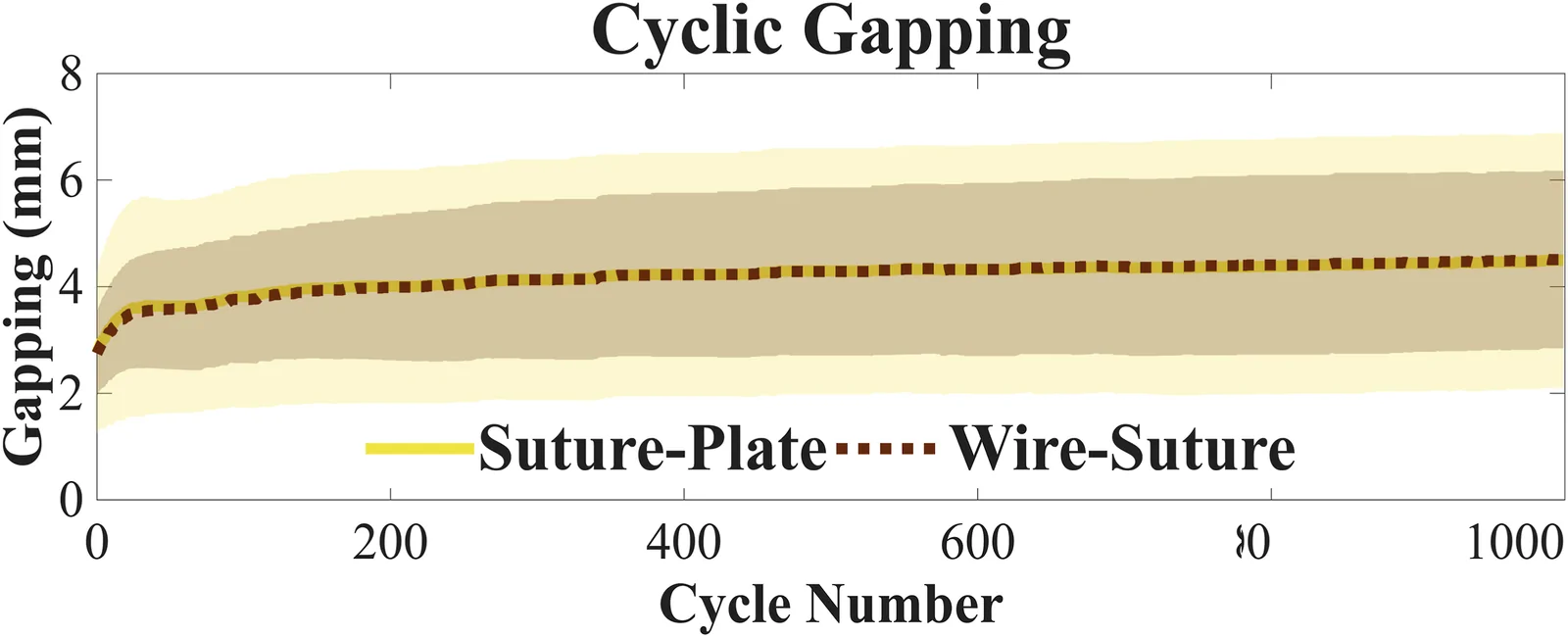
Construct gapping during cyclic testing. There were no differences in mean construct gapping between groups during cyclic testing. Data were interpolated between measured cycles to create continuous data for comparison. The solid yellow line represents the suture-plate construct mean and yellow cloud represents the standard deviation. The dotted brown line represents the wire-suture construct mean and brown cloud represents the standard deviation. No statistical differences were detected at any cycle.

**Table II:** Failure metrics (mean ± SD).

Variable	Suture	Wire	*P* value
Failure load (N)	647.5 ± 268.4	559.8 ± 253.6	.133
Failure displacement (mm)	11.2 ± 3.6	12.7 ± 9.6	.636
Construct stiffness (N/mm)	123.5 ± 28.9	102.8 ± 20.0	.033∗

*SD*, standard deviation.

∗ Statistically significant (*P* ≤ .050).

One specimen in each repair construct group, each in different pairs, did not fail prior to reaching the 1,050.0 N load cell limit (Table III). A secondary analysis where these specimens and their pair were excluded demonstrated no change in mean difference in construct stiffness; although the *P* value increased to *P* = .065. Neither failure load nor failure displacement changed. The most frequent failure mode in the suture-plate group was due to suture tearing (5/13, 38%), whereas the wire-suture group's most common failure mode was due to fractures through the bone tunnels created for the wires (10/13, 77%) (Table III). A secondary analysis showed that the suture-plate construct had a lower frequency of failure by bone tunnel fracture than the wire-suture construct (*P* = .021).

**Table III:** Failure modes (N [%]).

Failure mode	Suture	Wire
Humeral fracture	2 (15%)	0 (0%)
Suture tearing	5 (38%)	0 (0%)
Tendon rupture	3 (23%)	1 (8%)
Bone tunnel fracture	2 (15%)	10 (77%)
Osteotomy translation	0 (0%)	1 (8%)
No failure	1 (8%)	1 (8%)

Data for each specimen can be found in Supplemental Data, demonstrating the numeric differences in cyclic gapping at the specimen level despite no mean differences at each tested cycle.

## Discussion

This study compared the biomechanical properties of 2 previously described LTO repair constructs, a suture with a lateral plate^16^ and cerclage wire construct,^20^, ^26^ in the setting of total shoulder arthroplasty using stemless humeral components. There were no differences in cyclic gapping or failure load between the suture-plate and wire-suture constructs, suggesting there may not be differences in time-zero LTO fixation *in vivo*. However, the wire-suture construct displayed a lower construct stiffness and a primary failure mode of bone tunnel fracturing, suggesting that *in vivo*, the wire-suture construct may be less resilient to higher loads and may be at increased risk for fracturing at the bone tunnels.

In the present study, there were no differences in cyclic gap formation and load to failure between the suture-plate and wire-suture groups. This suggests that *in vivo*, there may be no perceptible differences in the time zero fixation of the LTO for each of these constructs. Additionally, the measured failure loads are supraphysiologic to the theoretical 250 N maximum internal rotation force of the subscapularis,^3^ but slightly lower than the approximate 700 N ultimate load of the tendon itself.^13^ Furthermore, 4 suture-plate specimens and 2 wire-suture specimens did not fail prior to either reaching the 1050 N load cell limit or to rupturing of the subscapularis tendon itself. With this understanding, it may be reasonable to conclude that the integrity of these constructs may only be challenged in rare, extreme overloading events such as in the case of a fall. Lastly, it is presumed that once healed, the bone would become the primary load-bearing entity rather than the construct.

However, the suture-plate group displayed an increased stiffness as compared to the wire-suture group, indicating that it takes more force to deform the suture-plate construct 1 mm. Theoretically, this increased stiffness is suggestive of a higher *in vivo* resilience to gap formation at increased loads. Tying the tension band sutures over a lateral plate likely distributed forces across a larger area of the greater tuberosity lateral cortex, reducing localized pressure and subsequent frequency of bone cutout. This broader load distribution could also be one origin of increased construct stiffness. While bone-to-bone healing is promoted by some micromotion, excess micromotion and fracture gapping increase fibrous tissue formation as opposed to new bone formation, therefore, resulting in poorer bone healing.^5^, ^15^, ^23^ The wire-suture repair construct failed with fracturing of the humeral bone tunnels 10 of 13 times as opposed to twice for the suture-plate repair construct. This indicates that patients with weaker bone are theoretically at higher risk for this type of failure *in vivo* when receiving an LTO repair with the wire-suture construct. However, definitive studies on the effect of bone quality on these constructs are not currently available in the literature.

Clinically, LTO repair with the wire-suture construct has a reported radiographic healing rate of 93% at a minimum of 2 years post-operatively when used with stemless components, with similar LTO union rates when compared with stemmed components.^20^, ^26^ Furthermore, patients saw significant improvements in forward elevation, external rotation, pain, and function.^26^ However, there is a reported 12.2% rate of radiographic evidence of broken cerclage wires and a 13.6% reoperation rate due to painful cerclage wires at an average of 2.5 years post-operatively.^26^ To make a direct clinical comparison in support of the observed biomechanical results, further studies should evaluate the clinical outcomes of patients undergoing stemless anatomic total shoulder arthroplasty with an LTO repair using the suture with a lateral plate construct. This construct may have the benefit of reduced need to reoperation for hardware removal without a loss of biomechanical strength.

The metal plate mounted against the humeral implant applied resistance to simulate glenohumeral contact forces (Fig. 5, B). The present study was able to achieve higher failure loads and displacements and no surgical neck fractures for the suture-plate construct when compared to a previous biomechanical study where the humeral implant was not resisted.^16^ Theoretically, this extra resistance reduced the stress on the surgical neck so it remains intact, allowing the construct to continue loading, increasing failure load and to better analyze the constructs' biomechanical properties. While a surgical neck fracture is a possible post-operative complication, it is rare and likely a result of a fall postoperatively, not forces applied to the lesser tuberosity via the subscapularis. Additionally, as a result of increased failure loads and displacements, lower stiffnesses were seen. The differences in the suture-plate construct failure loads, displacement, modes, and construct stiffness between studies are likely irrelevant, as they occur at supraphysiological loads, higher than those seen by patients post-operatively.

This study contains limitations. First, *in vivo* forces of the human were not directly replicated by the forces applied to the LTO, which were supraphysiologic. Also, this study did not evaluate LTO healing over time, demonstrating a worst-case scenario for acute failure after surgery. Additionally, living bone is generally stronger than the cadaveric bone used, so the results for the magnitudes of applied force and displacement should be interpreted with caution. Both constructs utilized sutures looped onto the stemless component, consistent with the authors' (P.C., R.T., C.J., M.S.) surgical practices, and therefore, may not be generalizable to similar constructs that do not incorporate direct humeral component fixation. However, clinical outcomes are similar when an LTO repair construct utilizes component fixation, regardless of component type (ie, full vs. stemless vs. mini).^12^, ^20^, ^22^ The suture-plate construct in this study is very specific including transosseous sutures tied in a racking hitch stitch (Nice Knot) along with inverted mattress stitches tied over a lateral plate. The data from this study cannot be inferred to all suture repair constructs as the specific technique and addition of a plate is key to the biomechanical strength. Similarly, you cannot then infer any suture repair construct is as strong as a wire-suture construct, just this specific configuration. Finally, one study coauthor is a paid consultant for a device manufacturer used in this study. Although this may be a potential source of bias, this study did not specifically analyze hardware related to this disclosure and is applicable to similar devices regardless of manufacturer. For this study, the stemless humeral components were donated by the device manufacturer. Although the manufacturer had no role in the study design, implementation, or analysis, the potential for bias associated with industry support remains.

## Conclusion

In this controlled laboratory study, there were no differences in gap formation and failure load between the suture with a lateral plate and cerclage wire constructs, suggesting that there may be no differences in time zero fixation and environments for *in vivo* LTO healing rates may exist. However, the decreased stiffness and increased number of bone tunnel failures seen in the cerclage wire-suture construct suggest that sutures with plate constructs may be more resilient at higher loads, and in patients with weaker bone, lower the risk for fracturing of the bone tunnels. While clinical data are needed to definitively prove this observation, LTO repair with a suture and lateral plate construct may provide a biomechanically superior repair to a cerclage wire-suture repair in the setting of stemless anatomic total shoulder arthroplasty.

## Disclaimers:

Funding: Experimental supplies were donated by Exactech Inc. (Gainesville, FL).

Conflicts of interest: Robert Z. Tashjian is a paid consultant for Stryker; holds stock in CoNextions and Genesis; receives intellectual property (IP) royalties from Zimmer Biomet, Stryker, and Shoulder Innovations; receives publishing royalties from Springer; and serves on the editorial board of the *Journal of Shoulder and Elbow Surgery*.

Peter N. Chalmers is a paid consultant for DePuy and Advita; receives IP royalties from DePuy, Responsive Arthroscopy, and Advita; holds stock in TitinKM Biomedical and Cinch; receives research support from DePuy and the National Institutes of Health; and serves on the editorial board of the *Journal of Shoulder and Elbow Surgery* Reviews, Reports, and Techniques.

Heath B. Henninger receives research support from Smith + Nephew, Stryker, the Department of Veterans Affairs, and the National Institutes of Health, and serves on the editorial boards of the *Journal of Biomechanics and the Journal of Biomechanical Engineering*.

Christopher D. Joyce is a paid consultant for Zimmer Biomet.

Any additional authors, their immediate families, and any research foundations with which they are affiliated have not received any financial payments or other benefits from any commercial entity related to the subject of this article.

## Ethics declaration

The authors did not obtain informed consent from participants or their legal representatives. All tissue obtained from national tissue banks was de-identified prior to procurement.

## References

[1] Aliaj K., Henninger H.B., Tétreault-Paquin J.E., Getelman M.H., Donahue J.P. (2019). Biomechanics of an interlinked suture anchor rotator cuff repair in a human cadaveric model. JSES Open Access.

[2] Boileau P., Alami G., Rumian A., Schwartz D.G., Trojani C., Seidl A.J. (2017). The doubled-suture nice knot. Orthopedics.

[3] Bull A.M.J., Reilly P., Wallace A.L., Amis A.A., Emery R.J.H. (2005). A novel technique to measure active tendon forces: application to the subscapularis tendon. Knee Surg Sports Traumatol Arthrosc.

[4] Choate W.S., Kwapisz A., Momaya A.M., Hawkins R.J., Tokish J.M. (2018). Outcomes for subscapularis management techniques in shoulder arthroplasty: a systematic review. J Shoulder Elbow Surg.

[5] Claes L.E., Heigele C.A., Neidlinger-Wilke C., Kaspar D., Seidl W., Margevicius K.J. (1998). Effects of mechanical factors on the fracture healing process. Clin Orthop Relat Res.

[6] Del Core M.A., Cutler H.S., Ahn J., Khazzam M. (2021). Systematic review and network meta-analysis of subscapularis management techniques in anatomic total shoulder arthroplasty. J Shoulder Elbow Surg.

[7] Denard P.J., Noyes M.P., Lädermann A. (2018). A tensionable method for subscapularis repair after shoulder arthroplasty. JSES Open Access.

[8] Faul F., Erdfelder E., Lang A.-G., Buchner A. (2007). G∗Power 3: a flexible statistical power analysis program for the social, behavioral, and biomedical sciences. Behav Res Methods.

[9] Fishman M.P., Budge M.D., Moravek J.E., Mayer M., Kurdziel M.D., Baker K.C. (2014). Biomechanical testing of small versus large lesser tuberosity osteotomies: effect on gap formation and ultimate failure load. J Shoulder Elbow Surg.

[10] Gerber C., Pennington S.D., Yian E.H., Pfirrmann C.A.W., Werner C.M.L., Zumstein M.A. (2006). Lesser tuberosity osteotomy for total shoulder arthroplasty: surgical technique. J Bone Joint Surg Am.

[11] Gerber C., Yian E.H., Pfirrmann C.A.W., Zumstein M.A., Werner C.M.L. (2005). Subscapularis muscle function and structure after total shoulder replacement with lesser tuberosity osteotomy and repair. J Bone Joint Surg Am.

[12] Guerrero E.M., Morwood M.P., Kankaria R.A., Johnston P.S., Garrigues G.E. (2019). Shoulder arthroplasty using mini-stem humeral components and a lesser tuberosity osteotomy. Musculoskelet Surg.

[13] Halder A., Zobitz M., Schultz F., An K. (2000). Structural properties of the subscapularis tendon. J Orthopaedic Res.

[14] Heckman D.S., Hoover S.A., Weinhold P.S., Spang J.T., Creighton R.A. (2011). Repair of lesser tuberosity osteotomy for shoulder arthroplasty: biomechanical evaluation of the Backpack and Dual Row techniques. J Shoulder Elbow Surg.

[15] Kenwright J., Richardson J.B., Cunningham J.L., White S.H., Goodship A.E., Adams M.A. (1991). Axial movement and tibial fractures: a controlled randomised trial of treatment. J Bone Joint Surg Br.

[16] Kirkham M.S., Percin B., Christy K.B., Guy C.R., Chalmers P.N., Tashjian R.Z. (2026). Lesser tuberosity osteotomy repair in stemless anatomic shoulder arthroplasty: a biomechanical analysis of repair techniques. J Shoulder Elbow Surg.

[17] Krishnan S.G., Stewart D.G., Reineck J.R., Lin K.C., Buzzell J.E., Burkhead W.Z. (2009). Subscapularis repair after shoulder arthroplasty: biomechanical and clinical validation of a novel technique. J Shoulder Elbow Surg.

[18] Kullar R.S., Reagan J.M., Kolz C.W., Burks R.T., Henninger H.B. (2014). Suture placement near the musculotendinous junction in the supraspinatus: implications for rotator cuff repair. The Am J Sports Med.

[19] Levy J.C., DeVito P., Berglund D., Vakharia R., Moor M., Malarkey A. (2019). Lesser tuberosity osteotomy in total shoulder arthroplasty: impact of radiographic healing on outcomes. J Shoulder Elbow Surg.

[20] Moverman M.A., Da Silva A., Joyce C.D., Chalmers P.N., Tashjian R.Z. (2025). Two-year functional and radiographic outcomes of a convertible metaphyseal-based short humeral stem in anatomic shoulder arthroplasty: a comparison to stemless humeral components. JSES Int.

[21] Pataky T.C. (2010). Generalized n-dimensional biomechanical field analysis using statistical parametric mapping. J Biomech.

[22] Phillips C., Pasqualini I., Barros H., Menendez M.E., Horinek J.L., Ardebol J. (2023). Lesser tuberosity osteotomy healing in stemmed and stemless anatomic shoulder arthroplasty is higher with a tensionable construct and affected by body mass index and tobacco use. J Clin Med.

[23] Pilliar R.M., Lee J.M., Maniatopoulos C. (1986). Observations on the effect of movement on bone ingrowth into porous-surfaced implants. Clin Orthop Relat Res.

[24] Piper C.C., Horneff J.G. (2022). Management of subscapularis insufficiency after total shoulder arthroplasty. J Am Acad Orthop Surg.

[25] Ponce B.A., Ahluwalia R.S., Mazzocca A.D., Gobezie R.G., Warner J.J.P., Millett P.J. (2005). Biomechanical and clinical evaluation of a novel lesser tuberosity repair technique in total shoulder arthroplasty. J Bone Joint Surg Am.

[26] Sato E.H., Simister S.K., Christensen G.V., Cizik A.M., Moverman M.A., Joyce C.D. (2025). Two-year functional and radiographic outcomes following large lesser tuberosity osteotomy in stemless anatomic total shoulder arthroplasty: a case series. JAAOS - J Am Acad Orthopaedic Surgeons.

[27] Scalise J.J., Ciccone J., Iannotti J.P. (2010). Clinical, radiographic, and ultrasonographic comparison of subscapularis tenotomy and lesser tuberosity osteotomy for total shoulder arthroplasty. J Bone Joint Surg Am.

[28] Schrock J.B., Kraeutler M.J., Houck D.A., Provenzano G.G., McCarty E.C., Bravman J.T. (2016). Lesser tuberosity osteotomy and subscapularis tenotomy repair techniques during total shoulder arthroplasty: a meta-analysis of cadaveric studies. Clin Biomech (Bristol).

[29] Tashjian R.Z., Chalmers P.N., Joyce C.D., Asghar E.B., Henninger H.B. (2024). Biomechanical comparison of suture bridge rotator cuff repair with and without dermal allograft pledgets. J Shoulder Elbow Surg.

[30] Van den Berghe G.R., Nguyen B., Patil S., D'Lima D.D., Mahar A., Pedowitz R. (2008). A biomechanical evaluation of three surgical techniques for subscapularis repair. J Shoulder Elbow Surg.

